# A Dual-Task Paradigm Combining Physical and Cognitive Training in Mice: Application to Aging

**DOI:** 10.14336/AD.2024.0207-1

**Published:** 2024-02-07

**Authors:** Elpidio Attoh-Mensah, Antoine Huret, Marianne Leger, Gilles Loggia, Gerald Nee, Stacy Largilliere, Daniel Zuba, Chantal Chavoix, Pascale Schumann-Bard, Thomas Freret

**Affiliations:** ^1^Normandie University, UNICAEN, INSERM, COMETE, CYCERON, CHU de Caen, 14000 Caen, France.; ^2^University of Limoges, HAVAE, UR 20217, F-87000 Limoges, France.; ^3^Normandie University, UNICAEN, CHU de Caen, Department of Geriatrics, Caen, 14000, France.

**Keywords:** Physical Activity, Cognitive Training, Dual-Tasking, Animal models, Aging and Prevention

## Abstract

Physical Activity (PA) is often associated with better overall health status, especially in older adults. Numerous pieces of evidence indicate that PA would be more beneficial when applied in conjunction with Cognitive Training (CT) either simultaneously (i.e., in Dual-Task [DT]) or sequentially. Nonetheless, the underlying mechanisms of such benefits remain elusive. To help delve deeper into their understanding, we developed a cognitive-motor DT paradigm in young adult mice and subsequently tested its effect in old age. Three groups of young adults C57BL/6J mice (3.5 months of age; n=10/group) were required. They were given cognitive tasks, either alone (Control) or in combination with PA which was administered either sequentially (SeqT group) or simultaneously (DT group). Mice were trained in a touchscreen chamber: first on a Visual Discrimination (VD) learning task, then on its Reversal (RVD) which assesses cognitive flexibility alongside procedural learning. PA was given through a homemade treadmill, designed to fit in the touchscreen chambers and set at 9 m/min. Fourteen months later, we further evaluated the effects of PA administered in both DT and SeqT groups, on the performance of the now 19-month-old mice. When compared to SeqT and control groups, DT mice significantly displayed better procedural learning in both VD and RVD tasks as young adults. In the RVD task, this enhanced performance was associated with both poorer inhibition and motor performance. Finally, in 19-month-old mice, both DT and SeqT mice displayed better motor and cognitive performances than control mice. This new cognitive-motor DT paradigm in mice yields an interesting framework that should be useful for adapting DT training in aging, including providing knowledge on the neurobiological correlates, to get the most out of its benefits.

## INTRODUCTION

Regular Physical Activity (PA) is commonly linked to improved overall health [1]. Numerous studies have demonstrated the preventive and protective effects of PA against different pathologies including cardiovascular conditions, diabetes, and even mental health disorders [2,3]. Public health services worldwide have thus launched several guidelines to favor PA practice, with older adults receiving particular attention given their frailty state. Accordingly, observations indicate that older adults receiving exercise as part of their care are less prone to experiencing functional decline or mobility issues, including falls [4]. Besides, PA interventions have been shown to benefit gait and several cognitive functions in older adults [5]. Additionally, a reduced risk of developing dementia has been reported in individuals with high levels of PA as opposed to less physically active cohorts [6-8].

Quite interestingly, the aforementioned beneficial effects of PA can also be achieved through training focusing on other modalities/functions. Several studies have indeed reported functional benefits of Cognitive Training (CT) for instance [9-11]. In line with this observation, new care approaches have suggested the combination of PA and CT in a single protocol to drain more benefits. Indeed, combination of such modalities is thought to yield additive or potentially synergistic beneficial effects [12,13]. Most studies have reported more benefits associated with the simultaneous (i.e., in Dual-Task [DT]) than the sequential combination of PA and CT [14,15]. This is all the more relevant as DT training is increasingly recommended to prevent age-induced functional decline in older adults [16].

The observed efficacy of DT training in enhancing functional improvement appears to stem from the reduction in competing resources inherent to cognitive-motor interference [17]. According to DT theories, such interference and thus the challenge in concurrently executing two tasks, results from the brain's limited resources [18,19]. By minimizing the resource competition with a DT training, the tasks are presumed to undergo automation, ultimately resulting in an enhanced overall performance [17].

Current DT trainings in aging involve the following PA (walking, dancing, or performing various aerobic exercises) combined with CT based on tasks involving executive control, attention, memory, and visuospatial domains [20,21]. Given this extensive array of opportunities, it is challenging to determine the most suitable one for older adults.

To make such a determination accurately, it appears crucial to elucidate the intricate relationship between motor and cognitive tasks while executing a DT training. Consequently, attaining a deeper understanding of the neurobiological correlates of DT training would unquestionably clear the path. Unfortunately, little is known so far, except for a few studies involving neuroplasticity and cerebral blood flow changes [21-23].

Animal models could be a great help in deciphering these mechanisms at work. Indeed, numerous advantages exist for experimental studies with animal models in providing evidence (e.g., recording of the activity of a single neuron, inactivation and electrical stimulation of brain regions, neuropsychopharmacology, ...). Yet, cognitive-motor DT trainings with an animal model are still lacking as previous studies have mostly considered two cognitive tasks in their paradigms [24]. Indeed, in the absence of verbal communication, cognitive tests in animals are based on the evaluation of a motor response (movement or absence of movement in an area of the testing device, interaction with another conspecific/an object, ...), making it tricky to add a significant motor challenge during behavioral cognitive tasks, to build a DT condition. For this reason, most animal studies have focused on the benefits of combining PA and CT only sequentially [25,26]. Quite interestingly, some authors have recently used the animals' ambulatory speed during increasingly demanding cognitive tasks as a reflection of DT condition [27]. Nevertheless, since the analysis was restricted to spontaneous locomotor movement (rather than a response to a challenging motor condition), this attempt does not encompass a genuine DT condition that involves simultaneous motor activity and cognitive stimulation.

The main purpose of our study was to investigate the feasibility of a cognitive-motor DT paradigm in mice. To this end, we took advantage of the automated touchscreen device that requires limited motor activity from animals to complete cognitive tasks [28], providing the opportunity to create a DT condition with an additional challenging walking task. In a second objective, we assessed the effects of such a combination of PA and CT, on the cognitive and motor performance of the same mice in old age to evaluate the usefulness of this paradigm in the study of age-related cognitive decline.

## MATERIALS AND METHODS

### Animals

C57BL/6J young adult male mice (n=30, aged 3 months), purchased from Janvier Labs were group-housed by 6, in standard polycarbonate cages (37x23x18cm). They were maintained on a 12:12h light/dark cycle (light off at 7a.m.) at constant temperature (21°C) and humidity (55%). The regional ethics committee (Comité d'Ethique NOrmande en Matière d'EXpérimentation Animale, CENOMEXA, agreement number: 24526) approved all the experiments, in compliance with the European directive 2010/63/EU for animal experiments.

### Behavioral procedures

*Food restriction protocol.* We monitored body weight daily throughout the behavioral procedures. After a week of acclimatization to the facilities, mice were food-restricted to 80-95% of the free-feeding body weight to maintain motivation during experiments (Horner et al., 2013).

**Figure 1.**
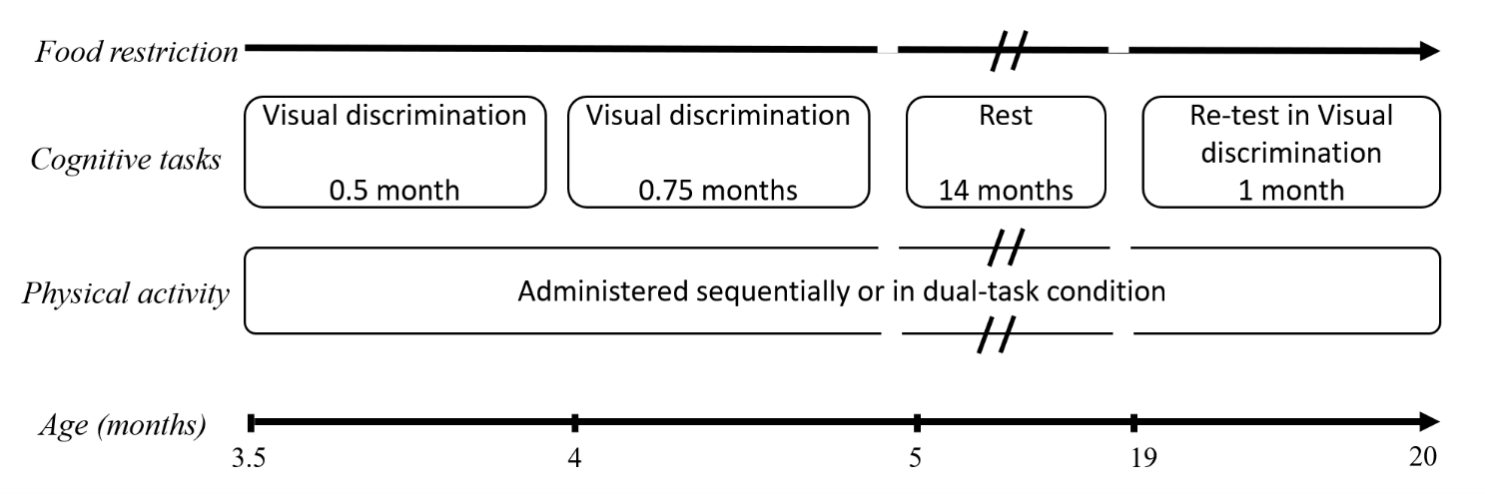
**Experimental procedure**. The tasks were performed in the three following groups: Cognitive task only (Control group), and cognitive task performed either sequentially (Sequential Tasks group, SeqT) or simultaneously (Dual-Task group, DT) with the treadmill walk physical activity (PA). All three groups were first trained successively in the visual discrimination task (VD) and its reversal (RVD). At the end of the RVD, mice were rested for 14 months and re-tested in the VD in the same group conditions to evaluate the effects of the two types of PA (SeqT and DT) in aged mice.

*Animal groups.* All experiments conducted are represented in Figure 1. Experiments started after two weeks of habituation to both cognitive and PA devices with execution of both tasks being equally rewarded to avoid task preference based on appetence. Three groups of mice were randomly constituted as follows: Control (Control), Sequential Tasks (SeqT), and Dual-Task (DT).

The control group was given only cognitive tasks. The SeqT group was additionally submitted to a treadmill walk in the touchscreen system (screen turned off), which was performed randomly either before or after cognitive tasks. A 5-minute walk was applied since we previously found that this was the average duration of locomotor activity for an animal during a session (sum of all trials). DT mice performed the PA during the cognitive tasks with the treadmill set to automatically start at the beginning of each trial within a session.

The treadmill was active as soon as visual stimuli were displayed on the touchscreen and stopped when the mouse elicited a response (either correct or incorrect).

*Cognitive tasks*. The Visual Discrimination (VD) task and its Reversal (RVD) were successively performed in touchscreen automated chambers (Campden Instruments Ltd., UK), as previously described [29].

During the VD task, mice were trained to discriminate between two visual stimuli (a fan [S-] and marbles [S+]), one of them being associated with a reward (S+). A mouse was rewarded with a liquid substance (creamy strawberry yogurt, Yoplait®, France) when the S+ stimulus was nose-poked. When the S- stimulus was nose-poked, a correction trial was given consisting of lighting the whole chamber and a timeout of five seconds. A session ended when the mouse completed 30 trials or after 60 min, whichever came first.

Mice were considered to have completed the VD task when they reached the criterion of 80% correct responses within 60 min, for 2 consecutive sessions.

Once a mouse reached this criterion at the VD task, it was passed on to the RVD task. The RVD testing procedure was the same as the VD one except for the inversion of the reward contingencies. Indeed, the previously rewarded stimulus S+ became the unrewarded stimulus S- (and *vice versa*). Each mouse was given 15 sessions, a sufficiently large number to reliably analyze the early stage at which cognitive flexibility is the most involved.

During both VD and RVD, the progression of animals to criterion across all sessions and the percentage of correct responses per session were examined. Besides, the perseverative index (number of correction trials/incorrect responses) across sessions was also analyzed in the RVD to reflect inhibition.

**Figure 2.**
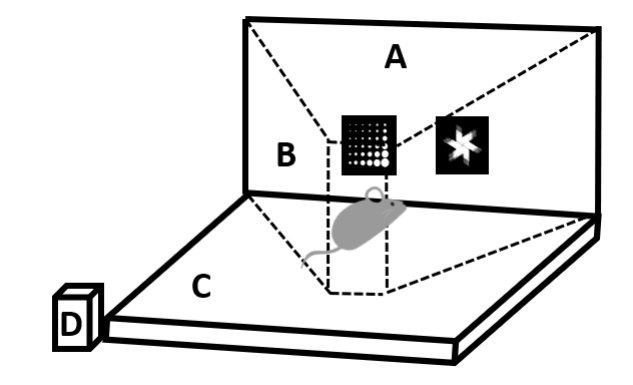
**Schematic representation of a mouse within the apparatus**. (**A**) Touchscreen with the two visual stimuli, marbles and a fan, (B) Walls of the automated chamber, (C) Treadmill belt in cotton tissue with two polyvinyl chloride pipes underneath and (D) Motor attached to the pipes to engage the rolling mechanism of the treadmill.

*Combination of physical activity and cognitive tasks.* To investigate the feasibility of a PA administered in combination, simultaneously or sequentially, with the previously described cognitive tasks, a homemade treadmill was specifically constructed to fit in the touchscreen chambers. This treadmill was composed of a motor attached to two rolling pipes in Polyvinyl Chloride (PVC) supported by a structure in aluminum. The PVC pipes were covered by a lane in cotton tissue which constitutes the treadmill belt (Fig. 2). The velocity of the treadmill was set at 9m/min in line with literature data [30]. This velocity corresponds to a challenging walking activity while being lower than the running speed used for fatigue protocols.

We considered ambulatory activity during cognitive tasks as an indicator of motor performance, as proposed by Hernandez and colleagues [27]. This motor performance was quantified by considering the impact of the three conditions (Control, SeqT, or DT) on the cumulative traveled distance needed to reach the criterion. To calculate this distance, we multiplied the sum of latencies to response elicitation during all trials needed to reach the criterion, by the walking speed.

In this calculation, for control and SeqT groups, in the absence of treadmill PA administered simultaneously with a cognitive task, we used a walking speed of 5.5 m/min consistent with the average walking speed of mice reported in the literature [31], while 9 m/min was used for DT group as the treadmill was set at this velocity.

*Physical activity and cognitive tasks delivered sequentially or simultaneously, in aged mice*. The three groups of mice were retested in the VD task fourteen months after completing the RVD, i.e. at the age of 19 months (a life stage previously described as displaying cognitive deficit in mice including in VD tasks - [32,33]). Mice were given 25 sessions of the VD task and were stopped when reaching the criterion. Of note, given the average 27% mortality rate observed in all groups (in agreement with the life span curve for this strain), the number of mice per group was 6 or 7 at the beginning of the retest.

### Data Analysis

All data are expressed as mean ± Standard Error Mean (SEM). The graphs were made using GraphPad Prism 8.0® (GraphPad Software, Inc., San Diego, CA) and analyses were carried out with SPSS 25.0® software (IBM; Armonk, NY, USA). The sequential Bonferroni correction method accounted for *post-hoc* multiple comparisons.

We performed a log-rank Mantel-Cox test with a survival/completion analysis to examine the progression of animals to criterion across all sessions. For the VD task, as previously described [29], the percentage of correct responses per session was analyzed using Generalized Estimating Equations (GEE) to account for missing values. For the RVD task, we focused on the analysis of the early stage (*i.e.*, sessions before control mice performance exceeded 50% of correct responses, where cognitive flexibility is the most involved). In the absence of missing values, we performed a two-way Analysis Of Variance with Repeated Measures (RM ANOVA) to analyze the percentage of correct responses and the perseverative index.

We analyzed motor performance through one-way ANOVA to compare the cumulative traveled distance needed to reach the criterion. Two mice (one in the Control and one in the ST group) did not complete all the behavioral experiments in young adults and were thus excluded from the statistical analysis.

## RESULTS

### Performance in young adult mice (3.5 months)

**Figure 3.**
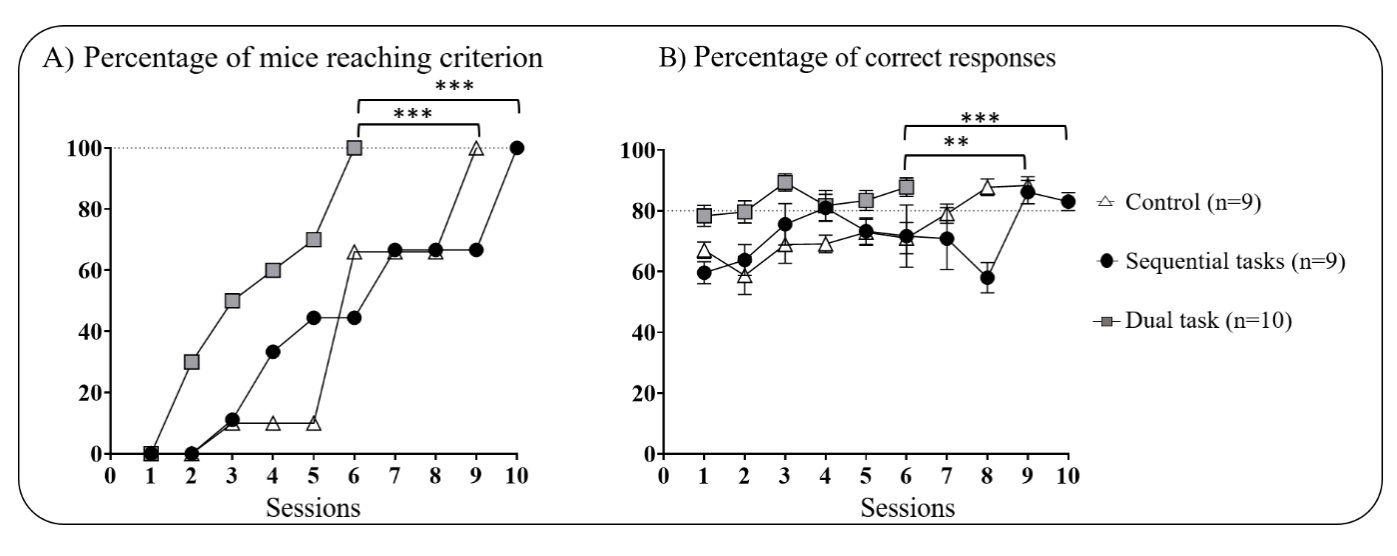
**Performances on the visual discrimination task in adult mice (3.5 months)**. (**A**) Progression of mice to criterion across sessions, and (B) Mean percentage of correct responses across sessions, **p<0.01, ***p<0.001 using a log-rank Mantel-Cox regression and Holm’s Sequential Bonferroni correction method after a Generalized Estimating Equation. Data are expressed as mean ± SEM.

*Visual Discrimination task (VD*). There was a main group effect in the progression of mice to criterion (Mantel-Cox: χ^2 (df 2)^ =46.78, *p*<0.0001, Fig 3A) and DT mice reached the criterion faster than SeqT and control mice (*p*<0.001 for both). Accordingly, the percentage of correct responses across sessions also differed between groups (GEE: Wald’s χ^2 (df 2,25)^ = 35.28, *p*<0.001; Fig 3B), with better performance in DT compared to either SeqT (*p*<0.01) or control groups (*p*<0.001). All animal groups learned the task as demonstrated by the session effect (GEE: Wald’s χ^2 (df 2,25)^ = 1888.15, *p*<0.0001; Fig 3B). Thus, we observed an increase in the percentage of correct responses across sessions regardless of group condition with a session x group interaction (GEE: Wald’s χ^2 (df 2,25)^ = 35.28, *p*<0.0001; Fig 3B). Finally, we noticed no difference between the performances of SeqT and control groups.

Of note, one-way ANOVA analysis did not reveal any intergroup differences in motor performances. All three groups traveled a similar distance to reach criterion (F_(2,25)_ = 2.61, *p*>0.1; mean values in meters: Control=195±80; ST = 117±42; DT = 145±82).

### Reversal visual discrimination (RVD)

**Figure 4.**
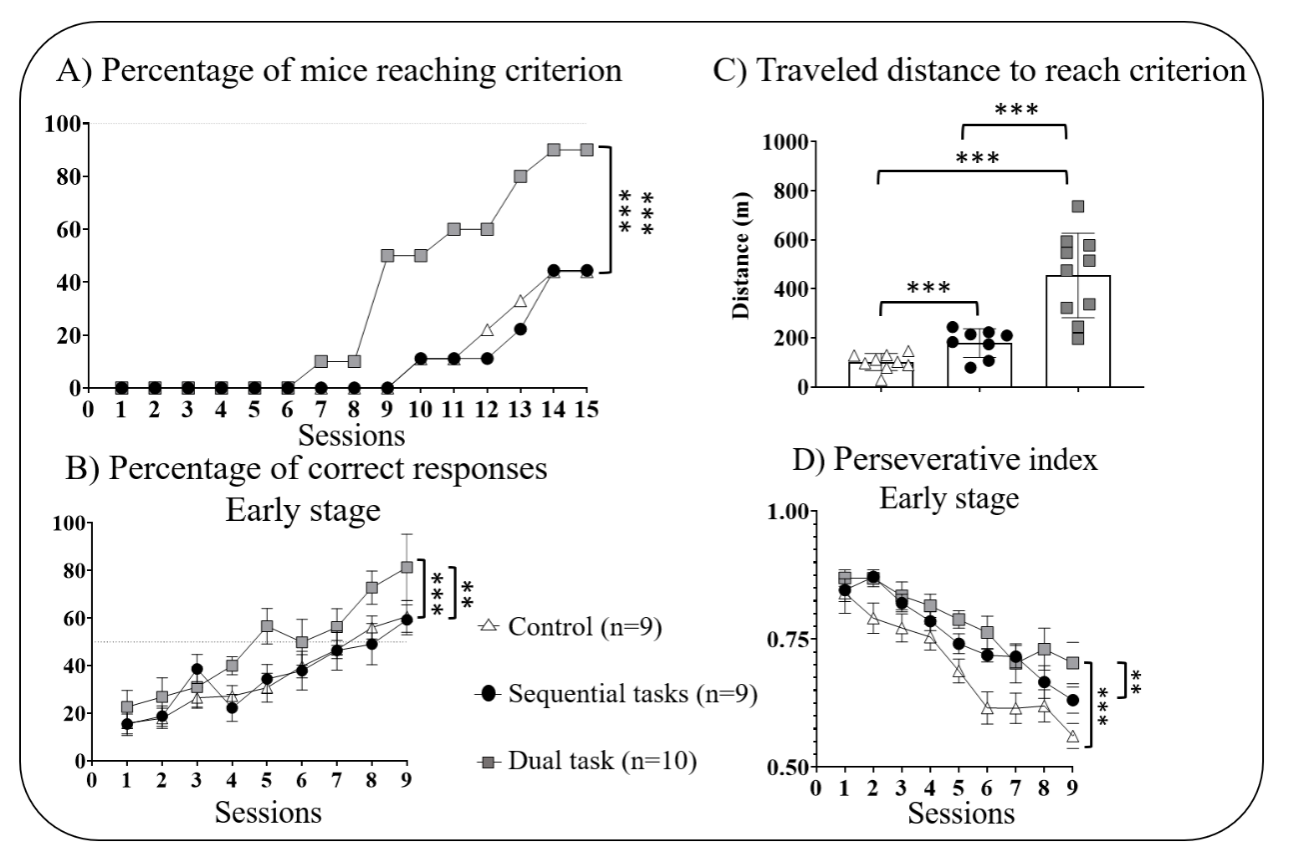
**Performances on the reversal of visual discrimination task in adult mice (3.5 months)**. (**A**) Progression of mice to criterion across sessions, (B) Percentage of correct responses across sessions (C), Mean cumulative traveled distance needed to reach criterion, and (D) Perseverative index. **p<0.01, ***p<0.001 using a log-rank Mantel-Cox regression and Holm’s Sequential Bonferroni correction method after a one-way or a two-way ANOVA with repeated measures. Data are expressed as mean ± SEM.

The analysis of the RVD performances also revealed a group effect for the progression of mice to criterion (Mantel-Cox: χ^2 (df 2)^ = 34.78, *p*<0.001; Fig 4A) and for the percentage of correct responses across sessions (RM ANOVA: F_(2,25)_ = 5.82, *p*<0.01; Fig 4B). For both parameters, the DT group displayed better performances than SeqT (*p*<0.01 and p<0.001, respectively) and control groups (*p*<0.001 for both).

A session effect (RM ANOVA: F_(2,25)_ = 12.32, *p*<0.001; Fig 4B) with no further session x group interaction (RM ANOVA: F_(2,25)_ = 4.78, *p*>0.1; Fig 4C) for the percentage of correct responses.

Conversely, some parameters were negatively impacted by the DT condition. Indeed, DT mice exhibited a higher perseverative index (poor inhibition) than both SeqT (*p*<0.001) and control groups (*p*<0.01) (RM ANOVA: F_(2,25)_ = 14.21, *p*<0.001; Fig 4D).

Furthermore, a group difference was also observed for motor performances (one-way ANOVA: F_(2,25)_ = 23.40, p<0.001; Fig 4C) with the DT group traveling more cumulative distance than both SeqT and the control group, to reach criterion (*p*<0.001 for both). Interestingly, the SeqT group traveled more distance than the control one (*p*<0.001).

### Effects of PA combined with CT, sequentially or in dual-task, in aged mice (19 months)

**Figure 5.**
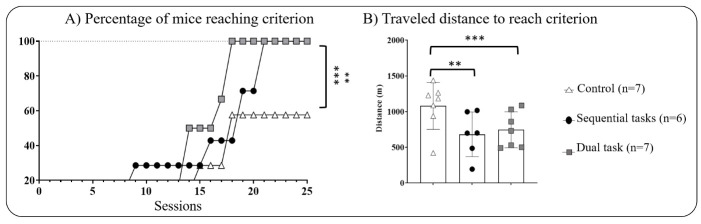
**Performances on the visual discrimination task in aged mice (19 months)**. (**A**) Progression of mice to criterion across sessions and (B) mean cumulative traveled distance needed to reach criterion during the re-test of visual discrimination task. **p<0.01, ***p<0.001 using a log-rank Mantel-Cox regression and Holm’s Sequential Bonferroni correction method after a one-way ANOVA, respectively. Data are expressed as mean ± SEM.

We found significant intergroup differences for the progression of aged mice to criterion (Mantel-Cox: χ^2 (df 2,17)^ =34.78, *p*<0.001, Fig 5A) with both DT and SeqT groups reaching criterion faster than the control group (*p*<0.001 and p<0.01, respectively).

However, we observed no significant intergroup difference for the percentage of correct responses across sessions (GEE: Wald’s χ^2 (df 2,17)^ =3.28, *p*>0.3) (Data not shown). Noteworthy, aged mice in both the DT and SeqT groups required approximately twice the number of sessions to reach the criterion as compared to their performance as young adult mice. Conversely, only 60% of the control mice were able to reach the criterion even after 25 sessions of testing.

Besides, both DT and SeqT groups traveled significantly less distance than the control group to reach the criterion (one-way ANOVA: F_(2,17)_ =34.78, *p*<0.001, *post-hoc* comparisons *vs* control, *p*<0.001 and p<0.01 for DT and SeqT groups, respectively, Fig 5B).

## DISCUSSION

The new cognitive-motor DT paradigm developed in the present study facilitated procedural learning during both VD and RVD in young adult and naïve mice (3.5 months). This was evidenced by DT mice reaching the criterion at a higher rate and achieving a higher percentage of correct responses (compared to SeqT and Control groups). We also found that DT condition with RVD hampered inhibition capacity and altered motor performance. Moreover, adult mice that have performed this DT and SeqT trainings further displayed better cognitive and motor performance than control mice at 19 months old.

In young adult and naïve mice engaging in this new DT paradigm, the enhanced discrimination performances in both VD and RVD within the DT group, as compared to both control and SeqT groups, aligns with some human studies [34,35]. Indeed, among the several theories proposed to explain how two tasks interact with each other during a DT paradigm, the cross-talk theory states that a better performance in DT than in a single task condition would originate from the synchronization of the two tasks that are guided by at least partly common neural pathways [34,35]. Interestingly, the iterative discrimination of stimuli, intrinsic to both VD and RVD is considered to be under the influence of procedural learning [36], which is known to leverage motor learning in both humans and animals. Therefore, it can be assumed that part of the neural pathways involved in such tasks were shared with those necessary for carrying out the motor task (PA), thus offering the possibility of pathway synchronization. Moreover, the observed deficiencies in both inhibition and motor performance during the RVD task within the DT group further reinforce the idea stated above. It is well-established that inhibition processes rely primarily on the prefrontal cortex, *i.e.*, on a neuronal pathway different from the one governing motor tasks [37], and that could explain the reduced performance in both tasks.

Indeed, the central capacity theory of dual-tasking [19], supports this assumption as it would indicate that brain resources were limited in processing effectively inhibition and motor-related tasks concurrently. Moreover, DT condition in the present paradigm affecting differently performances according to the cognitive task used (VD or RVD) is an interesting result. Notably, RVD requires the highest level of cognitive flexibility as rodents have to reverse their choice in regard to the rule learned during VD. The fact that, unlike with VD, DT condition with RVD negatively impacted both motor and cognitive performances could lead to the assumption that the complexity of the cognitive task can enhance the cognitive-motor interference in the present paradigm. This assumption is all the more compelling as the motor challenge was fixed in both aspects of DT condition (i.e., either with VD or RVD). Future studies should investigate whether increasing motor challenge also lead to the same results and consequently define the appropriate level of competing resources that both tasks should engage in to provide optimal benefits.

Furthermore, in addition to the fact that both cognitive and motor tasks were equally rewarded during habituation, several reasons could have accounted for limited prioritization between tasks. Indeed, because the treadmill covered the entirety of the touchscreen floor grid, mice were unable to avoid doing the motor task while executing the DT paradigm. In addition, as the learning criterion was reached faster in DT than is SeqT and control groups, the motor task was not prioritized over the cognitive task either.

When retested on VD 14 months later, we observed a performance decrease in control mice, as only 60% of them reached the criterion, even after 25 testing sessions, consistent with age-related cognitive impairment previously reported for this task [32,33]. Interestingly, we observed that both DT and SeqT groups showed better performance than the control group in cognitive as well as in motor tasks.

Without ruling out a possible explanation of the benefit of executing PA in both DT and SeqT conditions at old age, one can also genuinely hypothesize a long-lasting beneficial effect stemming from mice’s activity as young adults. While previous studies have reported the benefits of a sequential combination of PA and CT in animals [25,26], this is the first paper suggesting such a long-lasting positive impact of DT training in aged mice. Moreover, the fact that all aged mice in both DT and SeqT groups took twice as many sessions as young adults to reach the criterion mirrors findings in humans, where young adults (20-29 years old) outperformed older ones (60 to 74 years old and > 75 years old), indicating an age-related impact on dual-tasking [38].

Quite surprisingly, we were not able to highlight any difference in the performances of DT and SeqT groups in aged mice. This result could lead to the idea that there is no advantage of choosing DT training over a sequential combination of PA and CT. However, several reasons that might explain this absence of difference should be considered. First, while investigating the long-lasting effects of the combination of PA and CT, aged mice performed the task in the same conditions in which they were trained as young adults (i.e., Control, SeqT, or DT) to focus on the modalities trained. Henceforth, it is likely more cognitively demanding to perform a DT paradigm than a SeqT activity when aging, even though aged mice in the DT group were previously trained as young adults. This could explain why potentially more benefits resulting from DT training were not noticeable. To confirm this hypothesis, it would be interesting to perform crossover studies with each three-training condition in young and aged mice, as well as to investigate the positive transfer effect of the DT training on other functions with various behavioral tests. Second, due to the procedural nature of the VD task tested in aged mice, it can be assumed that the benefits of a DT training on performance in this task would be coarse given the known limited age-related effect on procedural abilities [39].

While being increasingly described as one of the preferred strategies to prevent functional decline or favor successful aging, DT training still lacks standards in the way it can or should be used (types of DT, intensity of task, duration of the training, ...) [40,41]. Therefore, DT training would likely be more beneficial if it was adapted to each individual.

Improving our knowledge of the brain regions and the neurobiological mechanisms involved in DT training would be a major asset in enhancing the beneficial effects. Therefore, the use of animal models appears insightful. Herein, we describe for the first time an animal model of a cognitive-motor DT paradigm while administrating simultaneously PA (*i.e.*, walking on a treadmill) and touchscreen-based CT (VD and RVD tasks) in mice. This new model deserves particular attention as it offers a high translational value, in particular, due to the devices used for both PA and CT. Indeed, both treadmills and touchscreen devices are already used in human studies, thus offering the advantage of efficient interspecies comparisons [28]. In addition, the touchscreen device can be coupled with several other techniques (*e.g.*, electrophysiology recording, optogenetics, ...), hence providing a unique setup to accurately dig into the underlying mechanisms of dual-tasking. Besides, the majority of DT-related performances in naive young adult mice, were specific to DT condition (as mice in SeqT and control groups performed similarly) which is an argument in favor of the utility of this paradigm in studying DT training. Furthermore, the fact that mice aged 19 months were still able to perform in the present DT paradigm and could even reach the criterion during the cognitive task, suggests that future studies can be conducted in old mice up to at least 19 months. This is a strength regarding studies in the aging field as DT paradigms are rare in animals [24], partly because they might be challenging to implement.

Some limitations to this study should be pointed out. First, the use of the cumulative traveled distance to reach the criterion as an index of motor performance can be criticized. Indeed, it only roughly overviews motor performance, and mostly its motivation component can bias results. Fortunately, intergroup differences in both body weight values and latency to behavioral response were limited (data not shown). Nevertheless, kinematic devices would afford proper motor performance measurements in future studies (stride length, stride variability ...). Second, one could also express concerns about the duration of the rest period applied (14 months) which led to a reduced size of experimental groups in aging animals (due to the expected 27% mortality rate given the life span curve of the mouse strain used) and prevented us from retesting mice in other behavioral tests than VD. While it's valid to approach our results in aged mice cautiously, it should be noted that we designed this study mostly to ensure that even old mice (19 months) were able to perform our newly developed DT paradigm. This information is a solid basis for future studies that aim to perform such DT training in middle-aged mice and to evaluate the effects on functional ability across aging. Ultimately, the complexity of implementing this paradigm, especially considering the investment in time and resources, is offset by its numerous advantages in elucidating the underlying mechanisms of dual-tasking during research.

In conclusion, this is the first study to develop a cognitive-motor dual-task paradigm in mice by combining physical activity and a touchscreen-based cognitive-stimulating task. This DT paradigm shares several characteristics with dual-tasks used in humans which allows in particular the evaluation of DT cost related to performance facilitation or depletion. This setup should significantly contribute to improving knowledge of the neurobiological correlates of dual-tasking by implementing various experimental studies. Such studies can help provide more standardized DT features to optimize the benefits of interventions based on DT training in aging.

Results regarding this new DT paradigm are all the more important as they would be more easily transferrable to humans, thanks to the interspecies comparison advantage of the touchscreen device.
